# Safety Profile of Statins for Post-Marketing Adverse Cardiovascular Events: A Real-World Pharmacovigilance Analysis

**DOI:** 10.2174/0118715303324204240905111835

**Published:** 2024-10-28

**Authors:** Jing Li, Junjie Gong, Ziyu Liu, Yuheng Liu, Anqi He, Zengguang Wang

**Affiliations:** 1 Department of Neurosurgery, Tianjin Medical University General Hospital, Tianjin, China;; 2 Key Laboratory of Post-Neuroinjury Neuro-Repair and Regeneration in Central Nervous System, Tianjin Neurological Institute, Ministry of Education and Tianjin, Tianjin, China;; 3 Tianjin Medical University, Tianjin, 300070, China;; 4 Tianjin Children's Hospital (Tianjin University Children's Hospital), Tianjin, 300134, China

**Keywords:** Statins, atorvastatin, adverse events, neurological, FDA Adverse Event Reporting System, real-world study

## Abstract

**Aims and objectives:**

The purpose of this study was to comprehensively evaluate the association of 3-hydroxy-3-methylglutaryl coenzyme A (HMG-CoA) reductase inhibitors (statins) with neurological adverse events using the US Food and Drug Administration Adverse Event Reporting System (FAERS) database, with the aim of guiding the rational use of statins.

**Methods:**

The number and clinical characteristics of adverse events (AEs) to statins in the FAERS database between 2012 and March, 2023, were extracted. Neurological AEs were defined by the system organ classes (SOCs) of “Nervous System Disorders (10029205)” and the corresponding PT. Disproportionality was calculated using the reporting dominance ratio (ROR), proportional reporting ratio (PRR), and information component (IC_025_).

**Results:**

Between January, 2012 and March, 2023, a total of 90,357 AEs were reported for the three statins (atorvastatin, resuvastatin, and simvastatin). The majority of reports on AEs came from the United States (n = 7284). A total of 8409 reports described neurological AEs following the use of the three statins, with atorvastatin accounting for more than half of the reports (n = 4430). The mean age of patients who developed neurological AEs was 55 years and older. The prevalence was similar in female patients (2230/4480) and male patients (1999/4480). Disproportionate analyses showed that at the SOC level, only the correlation between atorvastatin and neurological AEs suggested a positive signal (ROR: 9.77 (9.56-9.99); IC_025_: 3.28; PRR (χ^2^): 9.76 (16.07)) and in total, there were 32 PTs with a positive signal. The median time for neurological AEs was 71 days (IQR: 14-559 days), and the most common AEs were other serious effects (important medical event) (OT) (n = 2283) and hospitalization (HO) (n = 715).

**Conclusion:**

This study suggests that atorvastatin may be associated with an increased risk of neurological AEs. This study provides realistic evidence of the potential risk of statin-related adverse events.

## 1 INTRODUCTION

3-Hydroxy-3-methylglutaryl coenzyme A (HMG-CoA) reductase inhibitors (statins) are drugs commonly taken by patients with lipid disorders and are among the most commonly prescribed drugs in the world for the prevention of cardiovascular disease, particularly for the prevention of Coronary Artery Disease (CAD) events [1]. The success of statins has led to their increasingly widespread use, making them not only the world's best-selling drug but also the best-selling drug in history.

 However, any drug, regardless of benefit, carries a risk of Adverse Effects (AEs), and post-marketing surveillance often reveals AEs that may not have been apparent before. Several studies have reported that statins are associated with the development of muscle-related AEs and an increased risk of diabetes mellitus and hemorrhagic stroke [2]. In addition, the potential neurological AEs associated with statins are more pronounced in populations with primary prevention of CAD events [3]. Samaras *et al.* reported that statin use, especially long-term use, was associated with reduced cognitive performance [4]. Possible toxicity of statins on astrocytes has also been reported, which requires further mechanistic elucidation and rigorous experimental validation [5].

The FDA Adverse Event Reporting System (FAERS) database is a publicly available resource for adverse drug reaction data covering populations and multiple drugs worldwide [6, 7]. FAERS opens a new window for post-marketing clinical surveillance and risk assessment of drugs. In this study, the relationship between statins and neurological AEs in FAERS was evaluated using a well-established adverse event signal monitoring method, and the relationship between statins and neurological adverse events was explored and summarized from a pharmacovigilance perspective to provide a reference for clinical decision-making.

## 2 MATERIALS AND METHODS

### 2.1 Study Design and Data Sources

This is a retrospective study based on the FAERS public database. FAERS is an FDA-autonomous adverse event database designed to support the FDA's postmarketing safety surveillance of all approved drugs and therapeutic biologics. Each report in FAERS consists of seven data tables that include information on demographics and administrative information, drug use, dates of initiation and discontinuation of therapy, adverse events, indications, outcomes, and the source of the report [8, 9].

### 2.2 Drug Identification and Adverse Events (AEs)

In our study, the pharmacologic class of the target drug was statins. The following target drugs were selected: atorvastatin, rosuvastatin, and simvastatin, and all the reports containing generic and trade names were extracted.

In this study, we reviewed adverse reports from January 1^st^, 2012, to March 31^st^, 2023, regarding statins. Each report was categorized according to two factors: (1) only reports that listed statins as a “primary suspect” were selected for this study; (2) adverse events in FAERS were coded using preferred terms (PTs) in the Standardized Medical Dictionary for Regulatory Activities (MedDRA 26.0), and a PT can be associated with multiple system organ classes (SOCs) in MedDRA. To evaluate statin-induced neurotoxicity, we used the SOC “Nervous system disorders (10029205)” and the corresponding PT (MedDRA. Introductory Guide MedDRA Version 22.1, 2022). To exclude duplicate and conflicting data, assigned identifiers (unique IDs or case numbers) were reviewed on a case-by-case basis.

### 2.3 Signal Detection Methods

Pharmacovigilance studies use a case/non-case approach to analyze the risk of neurological AEs associated with statins. In this study, “cases” were defined as reports of nervous system disorders secondary to statins, whereas “non-cases” were defined as reports of all other statins-related AEs except nervous system disorders. In this study, we calculated the number of statins-associated AEs by calculating the number of statins-associated AEs. Moreover, we identified the adverse reaction signals by calculating ROR, IC_025_, and PRR Table **1**. The ROR is the odds of an event occurring with a drug compared to the odds of the same event occurring with all other drugs in the database [10]. PRR is a commonly used proportional dissonance measure in pairwise signal detection studies [11]. The information component (IC_025_) is a measure of the strength of quantitative dependence between a specific drug and a specific adverse drug reaction [12-14].

These indices were calculated using a 2×2 column table. The onset of the adverse event was the date on which the adverse event occurred (EVENT_DT) minus the date on which the medication was started (START_DT). We removed reports with data entry errors (EVENT_DT earlier than START_DT) and inaccurate data entries. In this study, R version 4.1.0 was used for the data processing and analysis described previously.

## 3 RESULTS

### 3.1 Descriptive Analysis

**Fig. (1):**
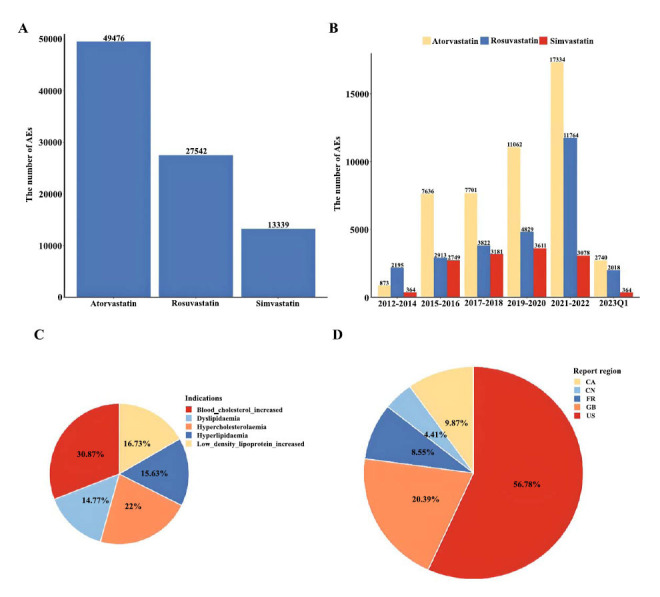
The descriptive analysis of clinical characteristics of patients with statins. (**A**) Between 2012 and March 2023, the number of AEs were reported for the three statins; (**B**) the number of AE reports per two-year period for three statins; (**C**) The main indications for these three statins; (**D**) The source of AE reports for three statins.

Between 2012 and March, 2023, a total of 90,357 AEs reports were reported for the three statins. Atorvastatin was the most affected class of statins (n=49,476, 54.76%), followed by rosuvastatin (n=27,542, 30.48%), while simvastatin was the least (n=13,339, 14.76%) (Fig. **1A**). Most AEs were reported between 2021 and 2022 (n=32176; 36.47%) (Fig. **1B**). The demographic and clinical characteristics of patients with statins in the FAERS are summarized in Table **2**. These reports involved more female patients (female: n=8520, 47.11%; male; n=7643, 42.26%). The largest number of patients were in their 60s (n=9162, 48.97%). The main indications for statins were increased blood cholesterol, hypercholesterolaemia, low-density lipoprotein increase, hyperlipidaemia, and dyslipidemia (Fig. **1C**). More than half of the adverse outcomes were reported as other serious/important medical events (OT) (n=8738, 46.81%) and hospitalization (HO). In addition, death (DE) and life-threatening (LT) were reported in similar numbers. The majority of AEs were reported from the United States (n=7284; 54.24%), followed by the United Kingdom (n=2616; 19.48%), Canada (n=1266; 9.43%), France (n=1097; 8.12%), and China (n=566; 4.21%) (Fig. **1D**). Healthy professionals and consumers appeared to be the most common spontaneous reporters compared to other reporters.

### 3.2 Description of Neurological AEs

We pooled the reports of neurological AEs to statins and summarized the clinical characteristics of the patients, which are presented in Table **3**. The incidence appeared to be the same in women (2230/4480, 49.78%) and men (1999/4480, 44.62%). The mean age of patients taking the three statins (atorvastatin, rosuvastatin, and simvastatin) was 55.86 ± 25.62, 58.80 ± 16.60, and 57.95 ± 20.02 years, respectively. Increased weight was found to be a significant factor in patients who developed neurological AEs (atorvastatin: 89.91 ± 37.48; rosuvastatin: 86.78 ± 32.45; simvastatin: 90.43 ± 35.33). To analyze the prognosis of neurological AEs associated with atorvastatin, we evaluated the adverse outcomes in the FAERS database. As mentioned in Table **3**, the adverse outcomes associated with neurological AEs were predominantly other serious events (important medical event) (OT) (2283/3578, 63.81%) and hospitalization (HO) (715/3578, 19.98%), with a fatal event rate of 2.39% (n = 447).

### 3.3 Disproportionate Analysis

Between 2012 and March, 2023, a total of 8409 statins-related neurological AEs were recorded in the FAERS database. Of these, atorvastatin accounted for more than half of the total number of reports (n = 4430, 52.68%), and the number of reports on rosuvastatin (n = 2583; 30.72%) was almost twice that of simvastatin (n = 1396; 16.60%). Based on the criteria of the three algorithms, we examined the signals of neurological AEs after statin use and presented the results in Table **4**. The results of the ROR, IC_025_, and PRR analyses showed that at this level of SOC, the risk of neurological AEs was higher only after atorvastatin use among the three statins (ROR: 9.77 (9.56-9.99); IC_025_: 3.28; PRR (χ^2^): 9.76 (16.07)).

**Fig. (2):**
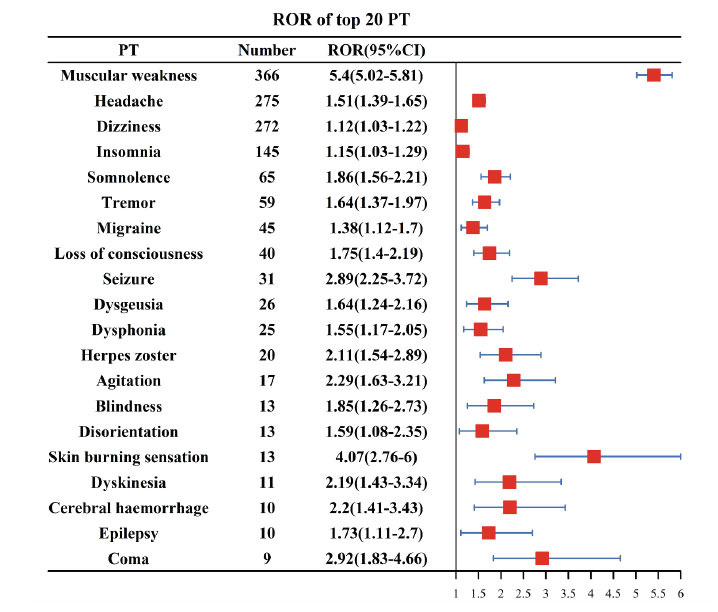
The ROR of top 20 PTs.

Since only the correlation between atorvastatin and neurological AEs was a positive signal, we next analyzed the signals at the PT level after atorvastatin use. A total of 32 significantly disproportionate PTs showed positive signals after atorvastatin administration, with the higher number of PTs being muscle weakness (n = 366), headache (n = 275), dizziness (n = 272), insomnia (n = 145), and somnolence (n = 65) (Fig. **2**). Among the AEs, migraine, loss of consciousness, seizure, dysgeusia, herpes zoster, and burning sensation of the skin did not appear in the drug labeling Table **5**.

### 3.4 Time to Onset of Neurological AEs Associated With Atorvastatin

**Fig. (3):**
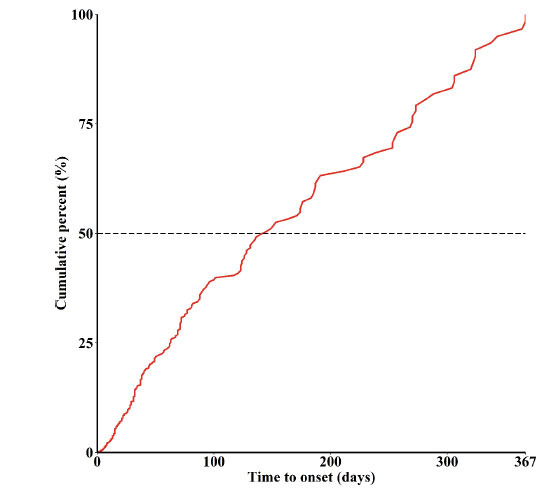
The time to onset of neurologic AEs after taking atorvastatin.

(Fig. **3**) depicts the time to onset of neurological AEs after taking atorvastatin for one year. Overall, the median time to onset of neurological AEs associated with taking atorvastatin was 71 days (IQR: 14-559 days). Furthermore, 37.02% (n=271) of the neurological AEs occurred in the first month of atorvastatin administration, and more than half (n=391, 53.42%) occurred in the first 3 months. Of note, we found that approximately 10% of neurological AEs occurred immediately after taking atorvastatin.

## 4 DISCUSSION

Adverse events are an important consideration in healthcare decision-making. As many interventions can cause serious adverse events, they can also lead to adverse outcomes, such as hospitalization, disability, and death, which can seriously affect patients' quality of life [15-18]. Therefore, physicians, pharmacists, and nurses need to be aware of adverse drug events. The FAERS database is a very important tool for post-marketing surveillance of drugs for pharmacovigilance studies [19].

In this study, we identified a total of 8409 neurological adverse events associated with statin use based on spontaneous reports in the FAERS database between 2012 and March, 2023. Disproportionality analysis showed that among the three statins, only atorvastatin use was associated with an increased incidence of neurologic adverse events. The novel findings of this study provide valid information on the safe use of atorvastatin and suggest that physicians should be cautious when prescribing atorvastatin and should inform patients in advance about the possibility of neurological adverse events, particularly muscle weakness, headache, dizziness, insomnia, and seizures. To our knowledge, this study provides the most comprehensive evaluation of the association between atorvastatin and the occurrence of neurological adverse events and also characterizes the clinical features of atorvastatin-associated neurological adverse events.

Existing studies have associated statin use with neurological disorders, including muscle symptoms, neuropathy, memory impairment, and other CNS changes, which is similar to our findings. First, both clinical trials and post-marketing surveillance have suggested that statins may be associated with adverse effects on muscle, liver, and kidney, particularly muscle complications, including pain, fatigue, weakness, and rhabdomyolysis [20]. Our study found that spontaneous reports on atorvastatin had the highest number of adverse events labeled as muscle weakness (n=366) and ROR=5.39, suggesting that muscle weakness may be the most important neurological adverse event of atorvastatin. Second, many studies and case reports have reported a significant association between statin use and the development of neuronal dysfunction. Golomb *et al.* reported a significant increase in the number of reports on motor neuron diseases, such as amyotrophic lateral sclerosis, with the use of rosuvastatin, pravastatin, atorvastatin, simvastatin, and lovastatin [21]. The results of this study showed that after taking atorvastatin, patients developed symptoms, such as dyskinesia and tremor. In addition, we found that multiple sclerosis relapse, multiple sclerosis, and seizure were also mostly reported as neuronal dysfunction disorders. This suggests that statins are likely to induce disorders related to neuronal dysfunction.

The Medicine and Healthcare Products Regulatory Agency (MHRA) found in European populations that the use of statins (atorvastatin, fluvastatin, lovastatin, pravastatin, rosuvastatin, and simvastatin) may have significant adverse effects on sleep quality. The agency also reminded drug companies to include sleep disturbances in the summary of product characteristics for all statins [22]. However, our study did not find a positive correlation between rosuvastatin and simvastatin and adverse neurological events, and sleep disturbances may occur only after taking atorvastatin. In recent years, attention has been drawn to the potential adverse neuropsychiatric effects of statins, particularly memory loss [23, 24], depression [25], suicide [26], and antisocial behavior [27-29]. Several case reports have suggested that statin use increases the risk of memory loss [30]. Golomb *et al.* reported that all patients experienced severe irritability manifestations, including homicidal impulses, threatening others, road rage, fear of family members, and property damage while taking statins [31]. In our study, neuropsychiatric disturbances, such as agitation, restlessness, and disorientation, were reported to be induced by atorvastatin use.

However, some studies contradict our current findings. The meta-analysis did not report that statins cause excessive violence [32]. Several studies suggest that statin use may not cause insomnia and sleep disturbances [33-35]. A meta-analysis of statin-associated memory loss found no prospective evidence of a neurocognitive risk from statins [36]. We hypothesize that these inconsistencies may be due to several reasons: First, because the manifestations of anger, irritability, and agitation were very mild, such neuropsychiatric events may not have been detected in the clinical trial and, consequently, detailed assessments were not performed; second, the investigators did not test for neurological disorders during the clinical trial phase; for example, statins have not been observed to increase rhabdomyolysis in clinical trials, although this is a recognized adverse event; and third, the clinical trial was conducted over a short period of time and the project ended without observing these adverse events. A study on the association between cholesterol-lowering medications and aggression test scores was conducted for only 3 months, but aggressive behavior was not significant until 1 year after medication [37].

Why does the use of atorvastatin lead to adverse neurological events? We hypothesize that this adverse outcome is related to dysregulation of lipid metabolism. Lipids are an integral part of myelin and synapses, and any factor that affects lipid homeostasis and metabolism in the brain has the potential to adversely affect brain function [38, 39]. Low serum cholesterol is associated with depression, suicidal tendencies, aggression, and antisocial behavior [31]. In primates, excessive lowering of cholesterol levels results in decreased central serotonin activity [40, 41], and excessively low central serotonin is associated with violent behavior [42]. Statin-induced neuropathy may be due to the fact that the lipid content of the brain is drastically reduced after administration of the drug, and the patient experiences discomfort. There are other mechanisms involved in the occurrence of adverse neurological events. The blood-brain barrier, composed of endothelial cells, pericytes, and astrocytes, protects the brain from harmful endogenous and exogenous substances [43]. Lipid peroxidation products may be potential biomarkers of oxidative stress status *in vivo* [44]. Oxidative stress induces many downstream biological events, such as activation of the JAK/STAT pathway, and mediates inflammatory responses to damaged neurons. More importantly, excessive lipid oxidation alters the physical properties of cell membranes of various types of cells in the blood-brain barrier and leads to neuronal injury and neuronal cell death through covalent modification of proteins and nucleic acids [45]. In addition to this, CoQ10 deficiency has been strongly associated with neurological damage. CoQ10 treatment rescued mitochondrial dysfunction and calcium dysregulation in COQ8A-ataxia Purkinje neurons and also rescued the viability, morphology, mitochondrial function, and Ca^2+^ buffering capacity of Purkinje neurons [46]. Statins are the first-line clinical lipid-lowering agents, particularly atorvastatin, which is widely used and has a significant lipid-lowering effect.

Currently, the labeling for atorvastatin only emphasizes the possible side effects of liver and kidney damage, myotoxicity, and hyperglycemia with the use of this drug. However, our study found that with continued use of atorvastatin, there is an increased risk of numerous neurological disorders, particularly headache, dizziness, insomnia, dysgeusia, dysphonia, and agitation; therefore, clinicians should not ignore the rare but serious neurological adverse events associated with this drug. Due to the extremely important cardiovascular benefits of atorvastatin, it is hoped that the results of this study will provide drug developers, physicians, pharmacists, and nurses with more comprehensive post-marketing safety information about the drug. Physicians should be aware of these potential adverse effects before prescribing atorvastatin and should explain the details of the complaints to their patients. Physicians and pharmacists should be especially cautious in elderly and heavy patients and conduct an appropriate risk-benefit assessment to determine whether treatment should be continued or modified.

Our study also has several limitations. First, the FAERS database has limitations specific to the form of reporting, such as overreporting, underreporting, reporting biases, and haphazard reporting. Second, the spontaneous reports lacked complete information about the patients, such as liver and kidney function, duration of drug intake, and risk factors, so findings from the spontaneous reporting system could only be used for qualitative studies; third, the results of the disproportionate analysis could only reveal associations and could not accurately infer a clear causal relationship between atorvastatin and neurological adverse events. Therefore, further experimental studies are still needed to be carried out.

## CONCLUSION

This study, based on the FAERS database, found a significant association between an elevated incidence of neurological adverse events and atorvastatin use; therefore, it is specifically recommended that physicians and patients be well-informed about the increased risk of neurological adverse events following atorvastatin prescriptions to ensure the safe use of the drug. The results of this study provide recommendations on the potential risks of atorvastatin and guidelines for the use of the drug.

## Figures and Tables

**Table 1:** Summary of formula applied for signal detection.

Formula	Equation	Criteria
ROR	ROR = (a/b)/(c/d)	95% CI > 1, N ≥ 2
	95%CI =e^ln(ROR)±1.96(1/a+1/b+1/c+1/d)^0.5^	
PRR	PRR=(a/(a+c))/(b/(b+d))	PRR≥2, χ2 ≥4, N ≥3
	χ2=Σ((O-E)2/E);(O=a,E=(a+b)(a+c)/(a+b+c+d))	
IC_025_	IC_025_=e^In(IC)-1.96(1/a+1/b+1/c+1/d)^0.5^	IC_025_>0

**Note: ** a: Number of reports on targeted adverse events occurring with the suspect drug;

b: Number of reports on off-target adverse events occurring with the suspect drug;

c: Number of reports on targeted adverse events occurring for all other drugs;

d: Number of reports on off-target adverse events for all other drugs.

**Table 2:** Demographic and clinical characteristics of patients with statins in the FAERS.

**Characteristic**	Atorvastatin	Rosuvastatin	Simvastatin	**Total**
**Gender**	-	-	-	-
Female	5449	1979	1092	8520
Male	4522	1868	1253	7643
Unknown	1420	481	23	1924
**Age group, y**	-	-	-	-
<18	62	23	16	101
18≤y≤60	2880	1093	578	4551
60<	5760	1953	1449	9162
Unknown	2779	1573	545	4897
**Indications**	-	-	-	-
Blood cholesterol increased	567	147	72	786
Hypercholesterolaemia	304	129	127	560
Low-density lipoprotein increased	410	8	8	426
Hyperlipidaemia	247	90	61	398
Dyslipidaemia	234	87	55	376
Unknown	7191	2197	973	10361
**Outcome**	-	-	-	-
Other serious/important medical event (OT)	5560	1945	1233	8738
Hospitalization (HO)	2149	875	679	3703
Disability (DS)	405	184	190	779
Death (DE)	275	94	78	447
Life-threatening (LT)	227	109	94	430
NA	2740	1502	327	4569
**Event year**	-	-	-	-
2012-2014	873	2195	364	3432
2015-2016	7636	2913	2749	13298
2017-2018	7701	3822	3181	14704
2019-2020	11062	4829	3611	19502
2021-2022	17334	11764	3078	32176
2023 quarter 1	2740	2018	364	5122
**Report region**	-	-	-	-
United States of America (US)	5280	1648	356	7284
United Kingdom of Great Britain and Northern Ireland (GB)	1513	321	782	2616
Canada (CA)	601	639	26	1266
France (FR)	704	196	197	1097
China (CN)	400	152	14	566
Unknown	79	482	40	601
**Reporter**	-	-	-	-
Healthy professional	5748	88	51	5887
Consumer	3274	93	24	3391
Other	517	4540	2536	7593

**Table 3:** Clinical characteristics of nervous system disorders related to statins reported in the FAERS database.

Characteristics (% of patients)	Atorvastatin	Rosuvastatin	Simvastatin	Total
**Gender**	-	-	-	-
Female	1230	657	343	2230
Male	1102	536	361	1999
Unknown	9	86	156	251
Average age (year), mean±SD	55.86±25.62	58.80±16.60	57.95±20.02	-
Weight (kg), mean±SD	89.91±37.48	86.78±32.45	90.43±35.33	-
**Indications**	-	-	-	-
Product used for unknown indication	294	63	45	402
Blood cholesterol increased	167	29	24	220
Hypercholesterolaemia	79	26	16	121
Blood cholesterol	51	8	8	67
Dyslipidaemia	48	7	6	61
**Outcomes**	-	-	-	-
Other serious (important medical event) (OT)	1240	621	422	2283
Hospitalization (HO)	539	213	173	715
Disability (DS)	202	87	83	372
Life-threatening (LT)	60	38	31	129
Death (DE)	33	21	15	69
Congenital anomaly (CA)	6	2	2	10

**Table 4:** Pharmacovigilance metrics for reported cases of nervous system disorders in statins.

Drugs name	Nervous system disorders (N)	ROR	IC_025_	PRR (χ2)
Atorvastatin	**4430**	**9.77 (9.56-9.99)**	**3.28**	**9.76 (16.07)**
Rosuvastatin	2583	1.02 (-0.006-1.05)	0.03	1.02 (0.04)
Simvastatin	1396	1.14 (1.10-1.19)	0.19	1.14 (0.25)
Total	8409	-	-	-

**Table 5:** Signals for the top 30 adverse events associated with nervous system disorders used by a number of reported cases.

pt	N	**ROR**	**95% CI**
Muscular weakness	366	5.39	5.01-5.81
Headache	275	1.51	1.39-1.64
Dizziness	272	1.12	1.03-1.22
Insomnia	145	1.15	1.02-1.29
Somnolence	65	1.85	1.56-2.21
Tremor	59	1.64	1.36-1.97
Migraine	45	1.37	1.11-1.69
Loss of consciousness	40	1.75	1.40-2.18
Seizure	31	2.89	2.24-3.71
Dysgeusia	26	1.63	1.24-2.15
Dysphonia	25	1.55	1.17-2.05
Herpes zoster	20	2.11	1.54-2.88
Agitation	17	2.28	1.62-3.21
Blindness	13	1.85	1.25-2.73
Disorientation	13	1.59	1.07-2.34
Skin burning sensation	13	4.07	2.76-6.00
Dyskinesia	11	2.18	1.43-3.33
Cerebral haemorrhage	10	2.20	1.41-3.42
Epilepsy	10	1.73	1.11-2.69
Coma	9	2.91	1.83-4.65
Hypersomnia	8	2.27	1.38-3.73
Restlessness	8	2.70	1.64-4.44
Altered state of consciousness	7	1.78	1.05-3.03
Multiple sclerosis	6	5.09	2.87-9.02
Multiple sclerosis relapse	6	7.86	4.44-13.92
Sciatica	6	1.79	1.01-3.17
Aphonia	5	1.91	1.02-3.58
Crying	5	4.14	2.21-7.75
Nervous system disorder	5	2.42	1.29-4.54
Carpal tunnel syndrome	4	2.36	1.17-4.77

## Data Availability

Data will be available on the FDA Adverse Event Reporting System (FAERS) (https://www.fda.gov/drugs/drug-approvals-and-databases/fda-adverse-event-reporting-system-faers).

## LIST OF ABBREVIATIONS

AEs: Adverse Events
SOCs: System Organ Classes
ROR: Reporting Dominance Ratio
HMG-CoA: 3-Hydroxy-3-Methylglutaryl Coenzyme A
HO: Hospitalization
